# Immunomodulatory effects of carbon ion radiotherapy in patients with localized prostate cancer

**DOI:** 10.1007/s00432-022-04194-9

**Published:** 2022-09-23

**Authors:** Wei Hu, Yulei Pei, Renli Ning, Ping Li, Zhenshan Zhang, Zhengshan Hong, Cihang Bao, Xiaomao Guo, Yun Sun, Qing Zhang

**Affiliations:** 1 Department of Radiation Oncology, Shanghai Proton and Heavy Ion Center, Fudan University Cancer Hospital, Shanghai, 201321 China; 2 Shanghai Key Laboratory of Radiation Oncology (20dz2261000), Shanghai, China; 3 Shanghai Engineering Research Center of Proton and Heavy Ion Radiation Therapy, Shanghai, 201321 China; 4 Department of Research and Development, Shanghai Proton and Heavy Ion Center, Fudan University Cancer Hospital, Shanghai, 201321 China; 5 Department of Radiation Oncology, Shanghai Proton and Heavy Ion Center, Shanghai, 201321 China

**Keywords:** Prostate cancer, Carbon ion radiotherapy, Peripheral immune cells, Immunomodulatory effect, Immune response

## Abstract

**Purpose:**

Radiotherapy is one of the main local treatment modalities for prostate cancer, while immunosuppressive effect induced by radiotherapy is an important factor of radiation resistance and treatment failure. Carbon ion radiotherapy (CIRT) is a novel radiotherapy technique and the immunomodulatory effect of CIRT provides the possibility of overcoming radioresistance and improving efficacy. The aim of this study was to assess the immune response evoked by CIRT in localized prostate cancer patients.

**Methods:**

Thirty-two patients were treated by CIRT combined with or without hormone therapy and peripheral blood samples were collected before and after CIRT. Investigation of peripheral immune cell frequency, proliferation, and cytokine expression was conducted by flow cytometry, real-time quantitative PCR and ELISA.

**Results:**

There were no significant differences in the frequencies of CD3 + , CD4 + , CD8 + T cells and NK cells after CIRT. CD4/CD8 ratio increased whereas B cells decreased. All lymphocyte subsets except regulatory T cells (Tregs) displayed increased proliferation and T cells exhibited increased functionality after CIRT, characterized by modestly increased cytokine secretion of TNF. Moreover, higher frequencies of Tregs were shown. Neither monocytic myeloid-derived suppressor cells (MDSCs) nor early MDSCs changed after CIRT. TGF-β1 gene expression decreased while IL-6 showed a non-significant trend towards a decrease. Both IL-10 gene expression and plasma TGF‐β1 level were unchanged.

**Conclusion:**

CIRT demonstrates the potential to elicit immune activation in localized prostate cancer patients, based on sparing lymphocytes, increased lymphocyte proliferation, enhanced T-cell functionality, together with limited induction of immunosuppressive cells and reduced expression of immunosuppressive cytokines.

**Supplementary Information:**

The online version contains supplementary material available at 10.1007/s00432-022-04194-9.

## Introduction

Prostate cancer is currently the second most common malignant cancer and the fifth leading cause of cancer death in males (Siegel et al. 2021). In China, the incidence of prostate cancer ranks the sixth among male malignant tumors (Xia et al. 2022). Radiotherapy is one of the main local treatments for prostate cancer. With the progress of radiotherapy planning system and imaging technology, the status of radiotherapy has gradually improved and become the preferred treatment strategy for patients who cannot tolerate surgery (Martin and D'Amico 2014).

As one of the important treatment modalities for prostate cancer, radiotherapy can not only elicit a direct tumor cell killing effect by inducing DNA strand breaks, but also induce the body to produce anti-tumor immune response, which has been confirmed in a series of studies on photon radiotherapy (Demaria et al. 2015; Deng et al. 2014; Kalbasi et al. 2013; Schaue et al. 2012). Radiation therapy can cause exposure of tumor antigens, which result in antigen presentation and T-cell activation (Demaria et al. 2015). Radiation therapy can also lead to the release of damage-associated molecular patterns (DAMPs) and induction of immunogenic cell death (Kalbasi et al. 2013). Furthermore, the release of a range of cytokines can be induced by radiotherapy to support anti-tumor immunity (Schaue et al. 2012).

However, much attention has been paid to the immunosuppression induced by photon radiotherapy. For example, photon radiotherapy of prostate cancer may produce a variety of chemokines, such as colony stimulating factor 1 (CSF1) and CXCL5, which promote the aggregation of MDSCs to tumors (Xu et al. 2013). MDSCs exert immunosuppressive function through various mechanisms. For instance, MDSCs can deplete L-arginine necessary for T-cell activation through high expression of arginase-1 and inducible nitric oxide synthase (iNOS) (Bronte and Zanovello 2005). In addition, nitric oxide, a product of L-arginine catabolism by iNOS, inhibits major histocompatibility complex (MHC) class II expression on antigen-presenting cells, thus blocking CD4 + T-cell activation and induces T-cell apoptosis (Gabrilovich and Nagaraj 2009). Liang et al. (Liang et al. 2017) proposed that monocytic MDSCs infiltration was an important mechanism of tumor radiation resistance, in which the expression of chemokine receptor CCR2 played a significant role. Moreover, Wu et al. (Wu et al. 2015) observed that local irradiation of prostate cancer may increase the infiltration of Tregs by promoting the secretion of immunosuppressive cytokine TGF-β, thus leading to resistance to photon radiation therapy. These immunosuppressive mechanisms and their induction of radiation resistance are likely to be important reasons for local recurrence and distant metastasis of prostate cancer. Therefore, a new therapeutic strategy to improve the efficacy of radiotherapy for prostate cancer is necessary to be explored.

Carbon ion radiotherapy (CIRT) is a novel and advanced radiotherapy technique. With unique physical and biological characteristics, CIRT is often adopted in tumors that are resistant to photon beams. It seems that CIRT has the potential advantage of inducing immune response. Carbon ion beams present a Bragg peak and provide a better dose distribution to the target volume, allowing the reduction of radiation injury to peripheral lymphocytes, which are essential for an effective immune response. As high linear energy transfer (LET) beams, carbon ion beams have higher relative biological effectiveness (RBE) and are more capable of causing serious DNA damage. The dsDNA fragments induced by CIRT are smaller and easier to leak into the cytoplasm through nuclear envelope ruptures. Then, the presence of cytosolic DNA after CIRT activates immune response via the cyclic GMP-AMP synthase/stimulator of interferon genes pathway (cGAS-STING pathway), which triggers type I interferon transcription (Durante and Formenti 2018).

Accordingly, the immunomodulatory effect of CIRT provides the possibility of overcoming radiation resistance and improving therapeutic effect. For patients with localized prostate cancer treated by CIRT, the improvement of 5-year progression-free survival and reduction of normal tissue toxicity have been truly confirmed in several clinical studies (Takakusagi et al. 2020; Mohamad et al. 2019; Sato et al. 2021), while the immune modulation of CIRT in prostate cancer has been poorly studied so far. The study of immune response evoked by CIRT is helpful to deepen the understanding of biological effects of carbon ion beams, explore potential predictive biomarkers and provide the basis for new treatment options that combine CIRT with immunotherapies. Thus, we initiated this prospective study to investigate the impacts of CIRT on peripheral immune cell composition, proliferation, and cytokine production of in localized prostate cancer patients.

## Materials and methods

### Patients and treatment

From May 2021 to March 2022, 32 patients undergoing definitive CIRT for localized prostate cancer were enrolled in this prospective study. Patient characteristics are shown in Table 1. The study was approved by the Institutional Review Board of Shanghai Proton and Heavy Ion Center and written informed consent was obtained from all participants. Patients were excluded if they had other uncontrolled primary malignancies, lymph nodes or distant metastasis, previous prostatectomy or pelvic radiotherapy, drug abuse or alcohol dependence, previous use of immunosuppressive therapies, or had infectious disease, HIV or hepatitis virus, syphilis, or even common cold.

**Table 1:** Patients’ characteristics

Characteristics	No. of patients	%
Age (years)		
< 60	3	9.4
≥ 60 and < 70	14	43.8
≥ 70 and < 80	9	28.1
≥ 80	6	18.8
Hormone therapy before CIRT (months)		
0	3	9.4
< 6	26	81.3
≥ 6	3	9.4
T stage		
T1	1	3.1
T2	25	78.1
T3	5	15.6
T4	1	3.1
Initial PSA (ng/ml)		
< 10	17	53.1
≥ 10 and ≤ 20	9	28.1
> 20	6	18.8
Gleason score		
6	8	25.0
7	12	37.5
≥ 8	12	37.5
Risk (NCCN)		
Low	1	3.1
Intermediate	15	46.9
High	10	31.3
Very high	6	18.8
Simultaneous integrated boost (SIB)		
Yes	15	46.9
No	17	53.1

All patients recruited were treated by CIRT combined with or without hormone therapy, based on their risk groups. According to the National Comprehensive Cancer Network (NCCN) guidelines, patients with low-risk prostate cancer needed no hormone therapy, intermediate-risk patients received continuous hormone therapy for at least 4–6 months, and high/very high-risk patients received continuous hormone therapy for 2–3 years. The recommended hormone therapy regimens were androgen blockade (bicalutamide), in combination with LHRH analogs (goserelin/leuprolide acetate). For all patients, the irradiation dose was 65.6 GyE in 16 fractions to the prostate and seminal vesicle (seminal vesicle was excluded for low-risk patients). Additional simultaneous integrated boost (SIB) up to 72 GyE to solid prostate tumors that were visible on multiparametric MRI and 68 Ga-PSMA PET/CT images was received by 15 of all the patients.

### Blood collection and peripheral blood mononuclear cell (PBMC) isolation

15 ml ethylenediaminetetraacetic acid (EDTA) peripheral blood samples were collected from the included patients within 4 h before beginning of the first fraction and 4 h after completion of the last fraction after informed consent. PBMCs were isolated by density gradient centrifugation using lymphocyte separation medium (Lymphprep™, STEMCELL Technologies), frozen in serum-free cell freezing medium (BAMBANKER™, NIPPON Genetics), and stored in -80℃ until use.

### Phenotypical characterization of PBMCs

Frozen PBMCs were thawed in Dulbecco’s Phosphate Buffered Saline. Antibody panels and staining protocols were established prior to the experiments. Each sample was incubated with fixable viability dye (Fixable Viability Stain 780, BD Biosciences) at room temperature for 10 min to rule out dead cells. After washed twice, Fc block (BD Biosciences) was added to significantly reduce potential non-specific antibody staining. Immune cell subset distribution was assessed by flow cytometry using two panels of antibodies against cell surface markers. Lymphocyte panel: CD3 PerCP-Cy5.5 (BD Biosciences), CD4 FITC (BD Biosciences), CD8 APC (BD Biosciences), CD25 BV421 (BD Biosciences), CD127 PE (BD Biosciences), CD19 APC-R700 (BD Biosciences), CD56 BV605 (BD Biosciences). MDSC panel: CD33 APC (BD Biosciences), CD11b FITC (BD Biosciences), HLA-DR PE (BD Biosciences), CD14 PerCP-Cy5.5 (BD Biosciences), CD15 BV421(BD Biosciences). For lymphocyte panel, after incubation of 30 min at 4 °C, cells were washed and permeabilized with fixation/permeabilization buffer (Transcription Factor Buffer Set, BD Biosciences) for 45 min at 4℃, followed by intracellular staining with Ki67 PE-Cy7 (BD Biosciences) for 45 min at 4℃ and two washes. Fluorescence minus one (FMO) controls were used for Ki67 PE-Cy7, CD11b FITC and HLA-DR PE.

### Intracellular cytokine staining

Frozen PBMCs were thawed and cultured in RPMI 1640 medium (Gibco) with 10% fetal bovine serum (Gibco), supplemented with 1% penicillin/streptomycin (Gibco) at 37 °C and 5% CO_2_. After overnight resting, cells were stimulated for five hours with Leukocyte Activation Cocktail with BD GolgiPlug™ (phorbol ester, phorbol 12-myristate 13-acetate, ionomycin and brefeldin A) (BD Biosciences) according to manufacturer’s protocol. The staining procedure was the same as the above part. Cells were incubated with Fixable Viability Stain 780 and Fc block successively, followed by surface staining containing antibodies CD3 PerCP-Cy5.5 (BD Biosciences) and CD8 APC (BD Biosciences). After washing and permeabilization, intracellular cytokine staining with IFN-γ PE (BD Biosciences) and TNF PE-Cy7 (BD Biosciences), as well as intracellular staining with CD4 BB515 (BD Biosciences) was conducted, followed by two washes. This method of staining for CD4 was adopted because phorbol 12-myristate 13-acetate can cause internalization and down-modulation of CD4.

### Flow cytometry and analysis strategies

All stainings were acquired using CytoFLEX S (Beckman Coulter) and data analysis was done using CytExpert (Beckman Coulter). MDSC staining was prioritized, followed by phenotyping and proliferation detection of lymphocyte subsets, then functional analysis of T cells. The gating strategy of lymphocyte subsets was as follows: lymphocytes (FSC-A vs SSC-A), singlets (FSC-A vs. FSC-H), living cells (Fixable Viability Stain 780 vs SSC-A). T cells were defined as CD3 + , B cells as CD3-CD19 + and NK cells as CD3-CD56 + . CD4 + , CD8 + T cells and Tregs (CD4 + CD25 + CD127-) were determined within CD3 + T cells. The gating strategy of MDSCs was as follows: PBMC (FSC-A vs SSC-A), singlets (FSC-A vs. FSC-H), living cells (Fixable Viability Stain 780 vs SSC-A). MDSCs were defined as HLA-DR-/low CD33 + CD11b + , within which monocytic MDSCs (M-MDSCs) were characterized as CD14 + CD15-, polymorphonuclear MDSCs (PMN-MDSCs) as CD14- CD15 + and early MDSCs (E-MDSCs) as CD14- CD15-. The percentage of Ki67 + cells was identified within each lymphocyte subset. Furthermore, intracellular cytokine production was assessed within the CD3 + CD4 + and CD3 + CD8 + T cells.

### Cytokine gene expression analysis

To examine IL‐10, TGF‐β1, and IL‐6 cytokine gene expression, total RNA was isolated from PBMCs using SteadyPure Quick RNA Extraction Kit (Accurate Biology) according to the manufacturer's instructions. Total RNA was quantified using a NanoDrop Lite spectrophotometer (Thermo Fisher Scientific). 1 µg of total RNA was reversely transcribed to produce cDNA using Evo M-MLV Mix Kit with gDNA Clean for qPCR (Accurate Biology) and then amplified using SYBR Green Premix Pro Taq HS qPCR Kit (Rox Plus) (Accurate Biology) following the manufacturer’s protocol. Real-time quantitative PCR was performed using QuantStudio™ 5 Real-Time PCR System (Applied Biosystems). Primer sequences of different genes were as follows: GAPDH forward, 5′- GTCTCCTCTGACTTCAACAGCG -3′; reverse, 5′- ACCACCCTGTTGCTGTAGCCAA -3′. IL-10 forward, 5′- TCAAGGCGCATGTGAACTCC -3′; reverse 5′- GATGTCAAACTCACTCATGGCT -3′. TGF‐β1 forward, 5′- CCCACAACGAAATCTATGAC -3′; reverse, 5′- CTGAGGTATCGCCAGGAA -3′. IL-6 forward, 5′- ACTCACCTCTTCAGAACGAATTG -3′; reverse 5′- CCATCTTTGGAAGGTTCAGGTTG -3′. IL-2 forward, 5′- CTCACCAGGATGCTCACATTTA -3′; reverse 5′- TCCAGAGGTTTGAGTTCTTCTTCT -3′. The relative mRNA level was normalized by the expression of GAPDH. The relative expression was determined by the 2^−ΔΔCT^ method.

### Determination of plasma cytokine concentration

Plasma was isolated from peripheral blood by centrifugation at 3000 rpm for 10 min. The Human TGF-beta 1 ELISA Kit (absin) was used to detect plasma levels of TGF‐β1 according to the manufacturer's instructions. The absorbance value was measured at a wavelength of 450 nm using Cytation 3 (BioTek), with a correction wavelength of 570 nm. The plasma samples were run in duplicate.

### Statistics

Data were expressed as mean ± standard error of the mean. Statistical analysis was performed with GraphPad Prism version 7.0.0 (GraphPad Software Inc., San Diego, CA, USA) and SPSS software Version 20.0 (SPSS Inc., Chicago, IL, USA). To evaluate statistical differences before and after CIRT, either paired Student’s *t*-test or Wilcoxon matched-pairs signed-ranks test was performed. The correlation between cytokine mRNA and protein levels was analyzed using Spearman’s rank correlation test. For comparison of differences between two CIRT groups, either two-tailed Student’s *t*-test or Mann Whitney test was used. *P* value < 0.05 was considered to be statistically significant. Significance was denoted as *p < 0.05, **p < 0.01 and ***p < 0.001.

## Results

### Changes in lymphocyte subpopulation composition after CIRT

**Fig. 1:**
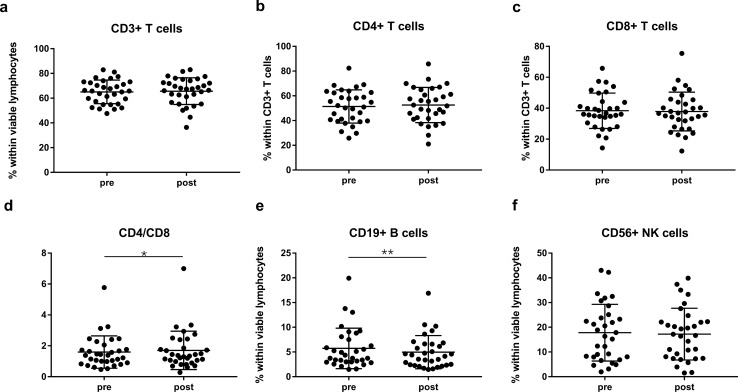
Effects of CIRT on lymphocyte subsets. **a** Percentage of CD3 + T cells within viable lymphocytes, **b**, **c** percentage of CD4 + and CD8 + T cells within CD3 + T cells, **d** CD4/CD8 ratio, and **e**, **f** percentage of CD19 + B cells and CD56 + NK cells within viable lymphocytes are shown before and after CIRT. *N* = 32 CIRT patients are included. Significant differences are depicted as *p < 0.05 and **p < 0.01

PBMCs were collected and the composition of lymphocyte subsets were assessed from 32 prostate cancer patients before and after CIRT. No significant difference was observed in the percentages of CD3 + T cells within viable lymphocytes, as well as CD4 + and CD8 + cells within CD3 + T cells (Fig. 1a-c). Interestingly, CD4/CD8 ratio increased after CIRT (pre: 1.59 ± 1.05, post: 1.74 ± 1.24; *P* = 0.0379) (Fig. 1d). The percentage of CD3-CD19 + B cells displayed a significant decrease post-CIRT compared to pre-CIRT (pre: 5.7 ± 4.1%, post: 5.0 ± 3.4%; *P* = 0.0042) (Fig. 1e). However, CD3-CD56 + NK cells did not change significantly after CIRT (Fig. 1f).

### CIRT increases lymphocyte subpopulation proliferation

**Fig. 2:**
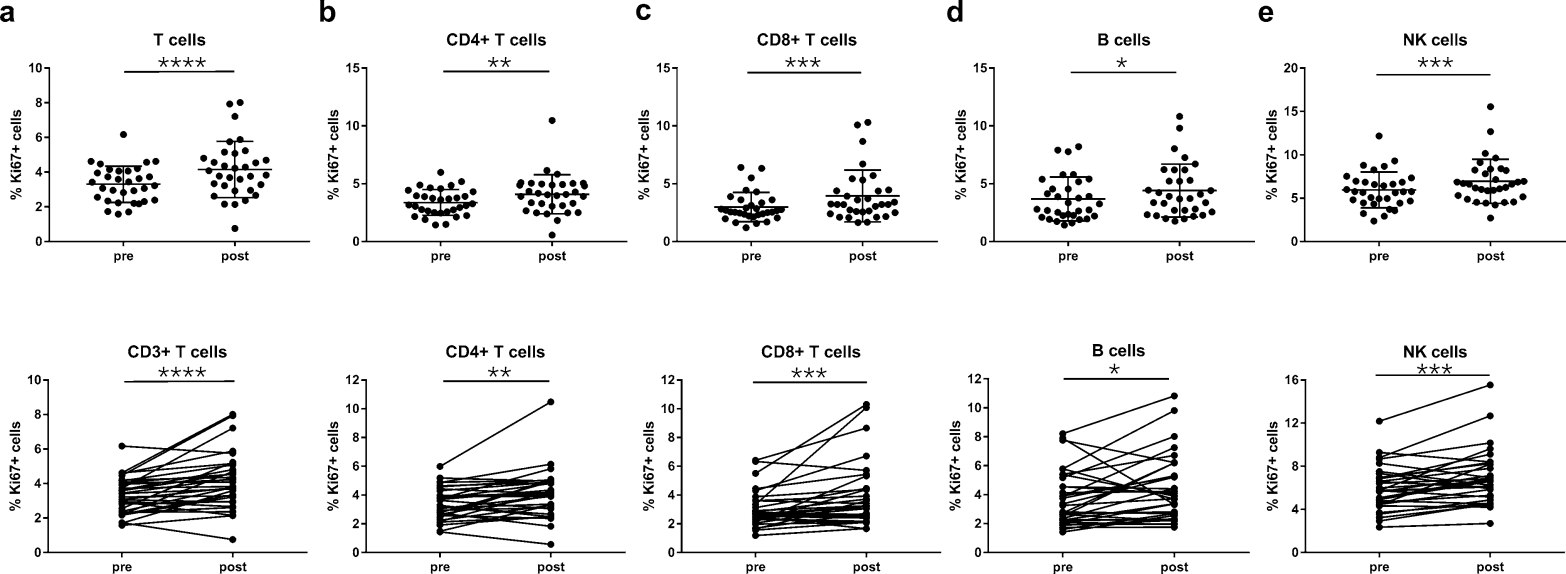
Proliferation of lymphocyte subsets. Percentages of Ki67 + cells within (**a**) CD3 + T cells, (**b**) CD4 + T cells, (**c**) CD8 + T cells, (**d**) B cells and (**e**) NK cells are shown before and after CIRT. *N* = 32 CIRT patients are included. Significant differences are depicted as *p < 0.05, **p < 0.01, ***p < 0.001 and ****p < 0.0001

To assess proliferation of lymphocyte subpopulations, intracellular staining of Ki67 was conducted. For all subsets, including CD3 + , CD4 + , CD8 + T cells, B cells and NK cells, similar dynamics with a significant increase in proliferation after CIRT were shown (CD3 + T cells: 3.3 ± 1.0% vs 4.2 ± 1.6%, *P* < 0.0001; CD4 + T cells: 3.4 ± 1.1% vs 4.1 ± 1.7%, *P* = 0.0038; CD8 + T cells: 3.0 ± 1.3% vs 3.9 ± 2.2%, *P* = 0.0004; B cells: 3.7 ± 1.9% vs 4.4 ± 2.3%, *P* = 0.0316; NK cells: 5.9 ± 2.1% vs 6.9 ± 2.6%, *P* = 0.0004) (Fig. 2a–e).

### Changes in T-cell function after CIRT

**Fig. 3:**
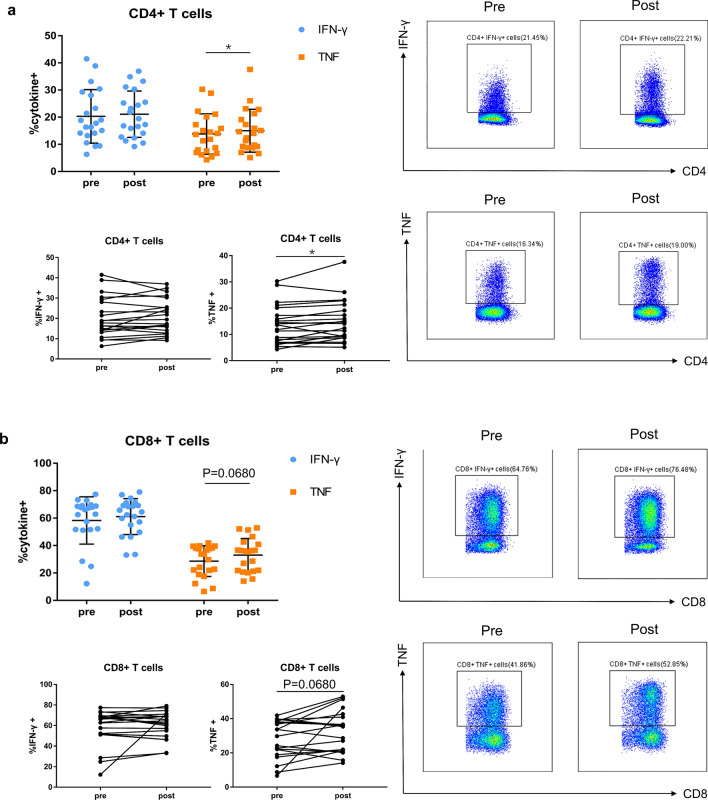
Function of T cells. Intracellular cytokine (IFN-γ and TNF) production of CD4 + (**a**) and CD8 + (**b**) T cells before and after CIRT. Representative gating for IFN-γ + and TNF + cells in the subset of CD4 + and CD8 + T cells is shown. *N* = 21 CIRT patients are included. Significant difference is depicted as *p < 0.05, and *p* < 0.1 is considered as statistical trend

To analyze T-cell functional properties, we performed intracellular cytokine staining of IFN-γ and TNF for CD4 + and CD8 + T cells after stimulating patients’ PBMCs for five hours. T-cell function analysis was performed in 21 of the 32 patients enrolled because of limited cell number of PBMCs. We found that cytokine IFN-γ production in the CD4 + and CD8 + T cells remain unchanged post-CIRT compared to pre-CIRT status (Fig. 3a–b). Contrasting with IFN-γ, cytokine TNF demonstrated a significant increase in CD4 + T cells (pre: 13.8 ± 7.4%, post: 15.0 ± 7.9%; *P* = 0.0462) and a non-significant increasing trend in CD8 + T cells (pre: 28.6 ± 11.2%, post: 33.1 ± 12.1%; *P* = 0.0680) after CIRT (Fig. 3a–b).

### Effects of CIRT on frequencies of Tregs and MDSCs

**Fig. 4:**
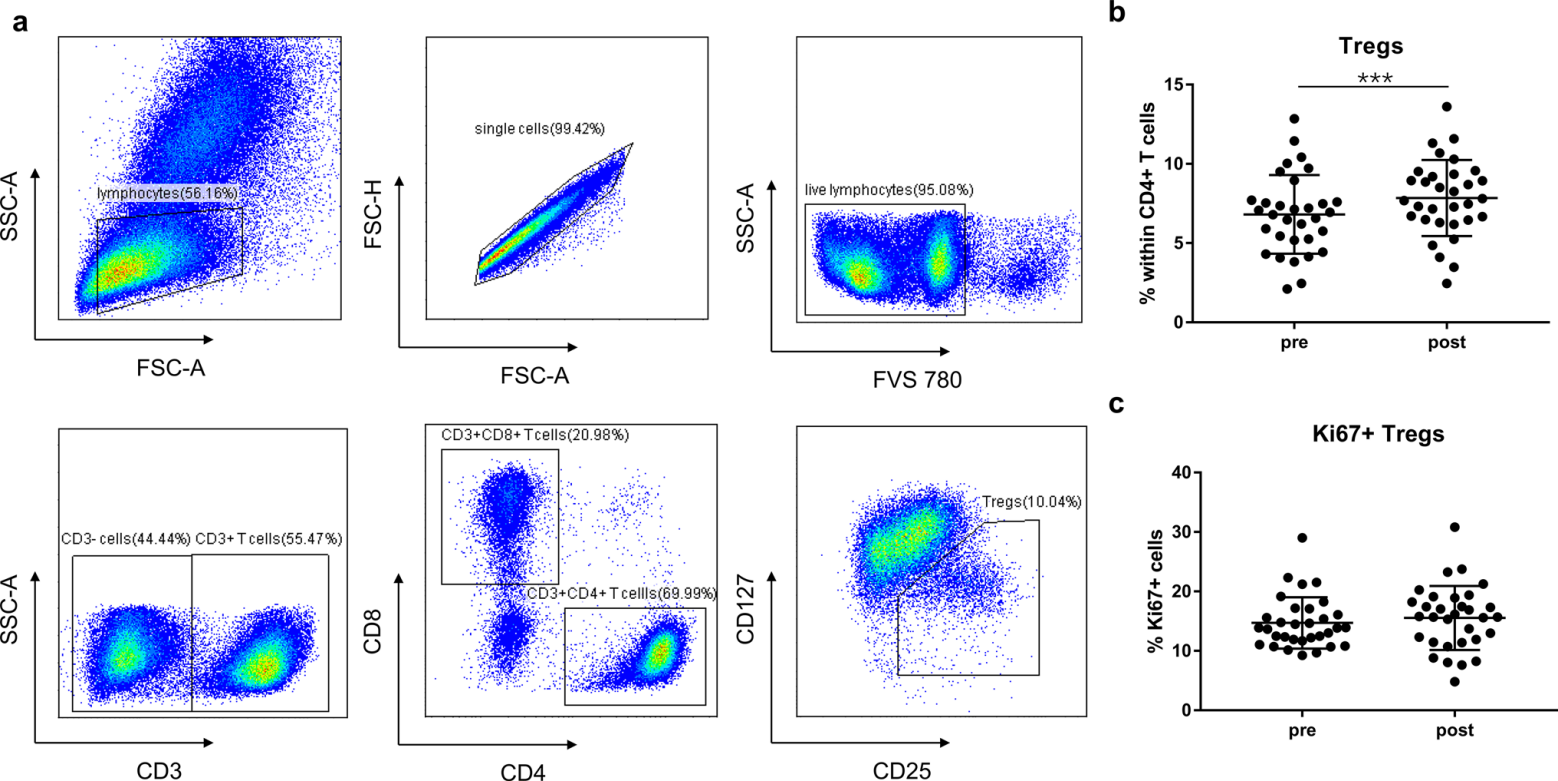
Effects of CIRT on Tregs. **a** Gating strategy of Tregs is shown. **b** Percentage of Tregs within CD4 + T cells, and **c** percentage of Ki67 + cells within Tregs are shown before and after CIRT. *N* = 32 CIRT patients are included. Significant difference is depicted as ***p < 0.001

To determine the effect of CIRT on immunosuppressive cell subsets, we also detected the changes of Tregs and MDSCs before and after CIRT. Tregs were defined based on surface markers as CD3 + CD4 + CD25 + CD127- (Fig. 4a) because previous data (Liu et al. 2006) showed that the majority of these cells were actually FoxP3 + . We found significantly higher frequencies of circulating Tregs post-CIRT when compared to pre-CIRT (pre: 6.8 ± 2.5%, post:7.8 ± 2.4%; *P* = 0.0003) (Fig. 4b). In contrast, the proliferation of Tregs was not affected after CIRT (Fig. 4c).

**Fig. 5:**
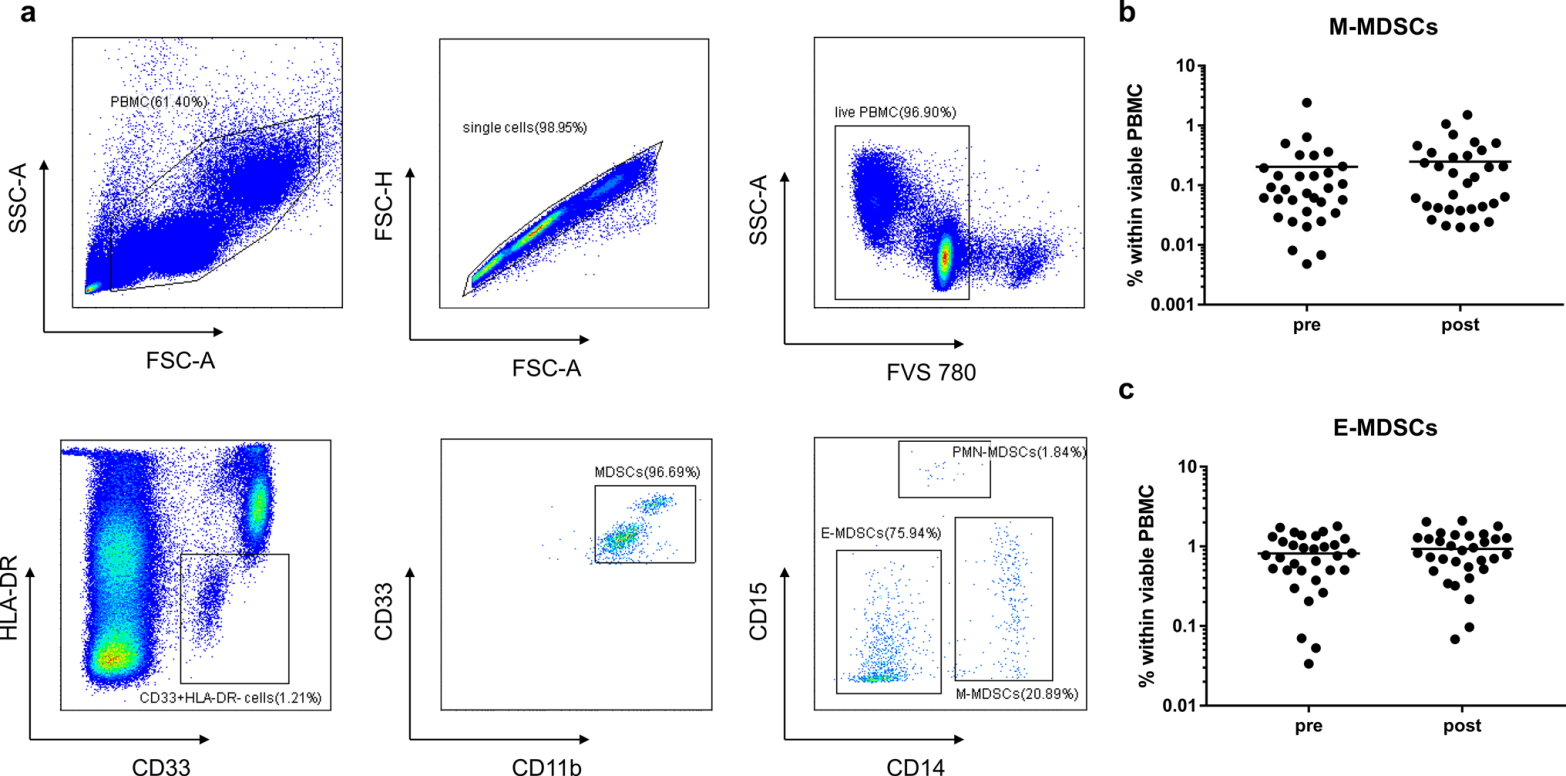
Effects of CIRT on MDSCs. **a** Gating strategy of MDSCs is shown. **b**, **c** Percentages of M-MDSCs and E-MDSCs within viable PBMCs are shown before and after CIRT. *N* = 32 CIRT patients are included

MDSCs were defined as HLA-DR-/low CD33 + CD11b + , which can be divided into three subtypes, namely PMN-MDSCs, M-MDSCs and E-MDSCs (Fig. 5a). As reported in the study (Idorn et al. 2014) and as we found during the experiment (data not shown), PMN-MDSCs were highly susceptible to freeze–thaw cycles, so we decided to focus on M-MDSCs and E-MDSCs. However, neither M-MDSCs nor E-MDSCs showed a statistically significant difference after CIRT (Fig. 4b, c).

### Influence on immunosuppressive cytokine expression after CIRT

**Fig. 6:**
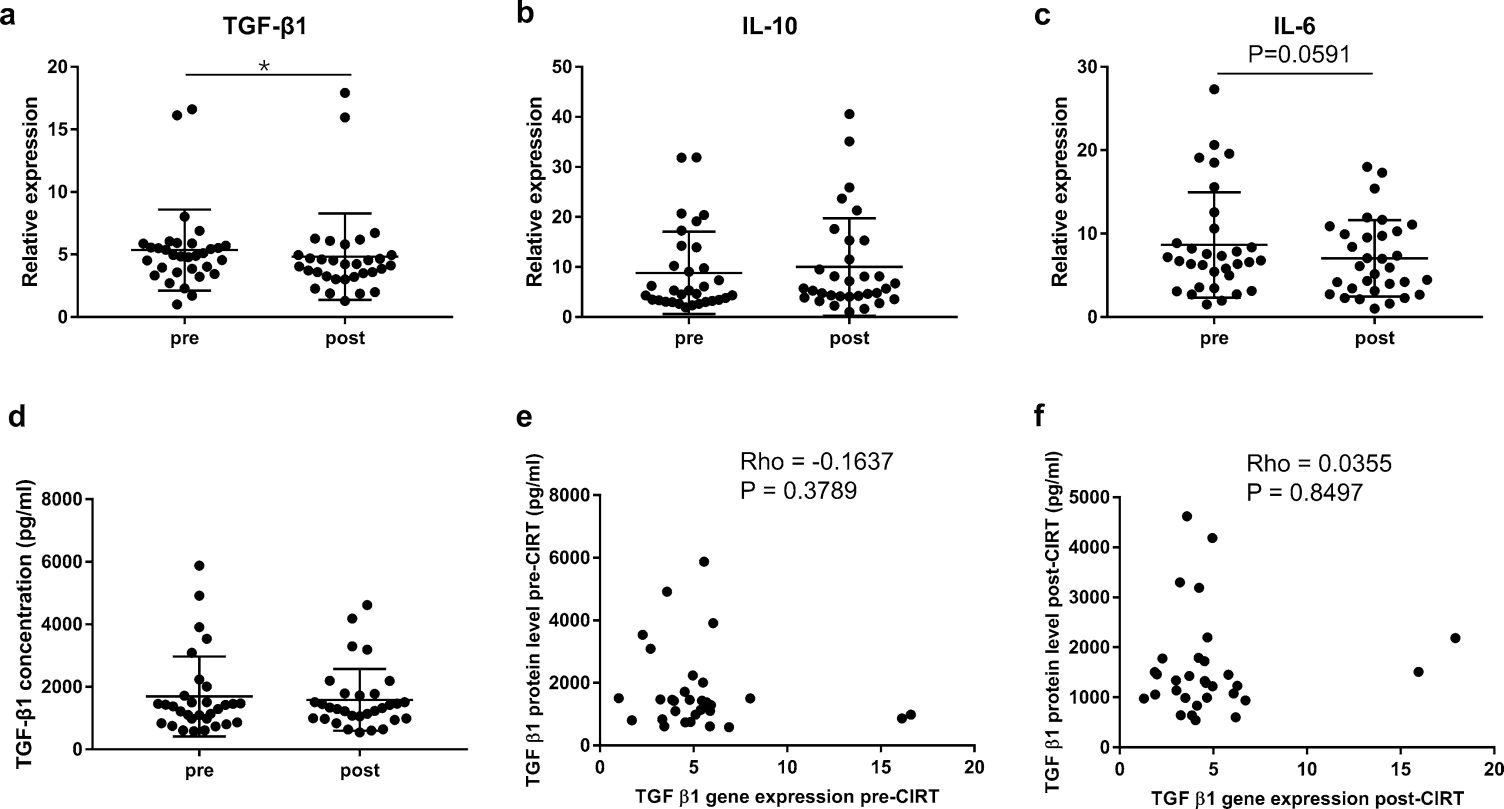
Cytokine mRNA and protein levels and correlations between mRNA and protein levels. Cytokine gene expression levels of (**a**) TGF-β1, (**b**) IL-10 and (**c**) IL-6 are shown before and after CIRT. *N* = 32 CIRT patients are included. (**d**) Plasma TGF-β1 concentration is shown before and after CIRT. *N* = 31 CIRT patients are included. (**e**, **f**) Correlations between gene and protein levels of TGF-β1 at pre-CIRT or post-CIRT timepoint. *N* = 31 patients are included. Significant difference is depicted as *p < 0.05

As TGF-β, IL-10 and IL-6 are key factors of MDSCs and Tregs mediated immunosuppression, we next evaluated these cytokines at mRNA levels in 32 prostate cancer patients. TGF-β1 gene expression level significantly decreased after CIRT (pre: 5.35 ± 3.24, post: 4.83 ± 3.46; *P* = 0.0310) (Fig. 6a), while no significant change occurred to IL-10 level (Fig. 6b). Also, there was a non-significant trend towards a decrease in the gene expression level of IL-6 after CIRT (*P* = 0.0591) (Fig. 6c). In terms of the essential role of IL-2 in the development and function of Tregs, we detected IL-2 gene expression level and found no significant variation (Supplementary Fig. 1) after CIRT.

Of the 32 patients enrolled, plasma was available in 31 patients treated with CIRT. We further conducted ELISA assay to detect TGF‐β1 level using plasma collected from the 31 patients. Surprisingly, we did not observe a significant difference to plasma TGF‐β1 level post-CIRT compared to pre-CIRT (Fig. 6d).

Moreover, we examined the correlation between TGF-β1 gene expression measured in PBMCs and protein levels measured in plasma of matched patients, and unexpectedly detected no significant correlation at pre-CIRT or post-CIRT timepoint (Fig. 6e–f).

### Irradiation dose impacts immune cell changes after CIRT

**Fig. 7:**
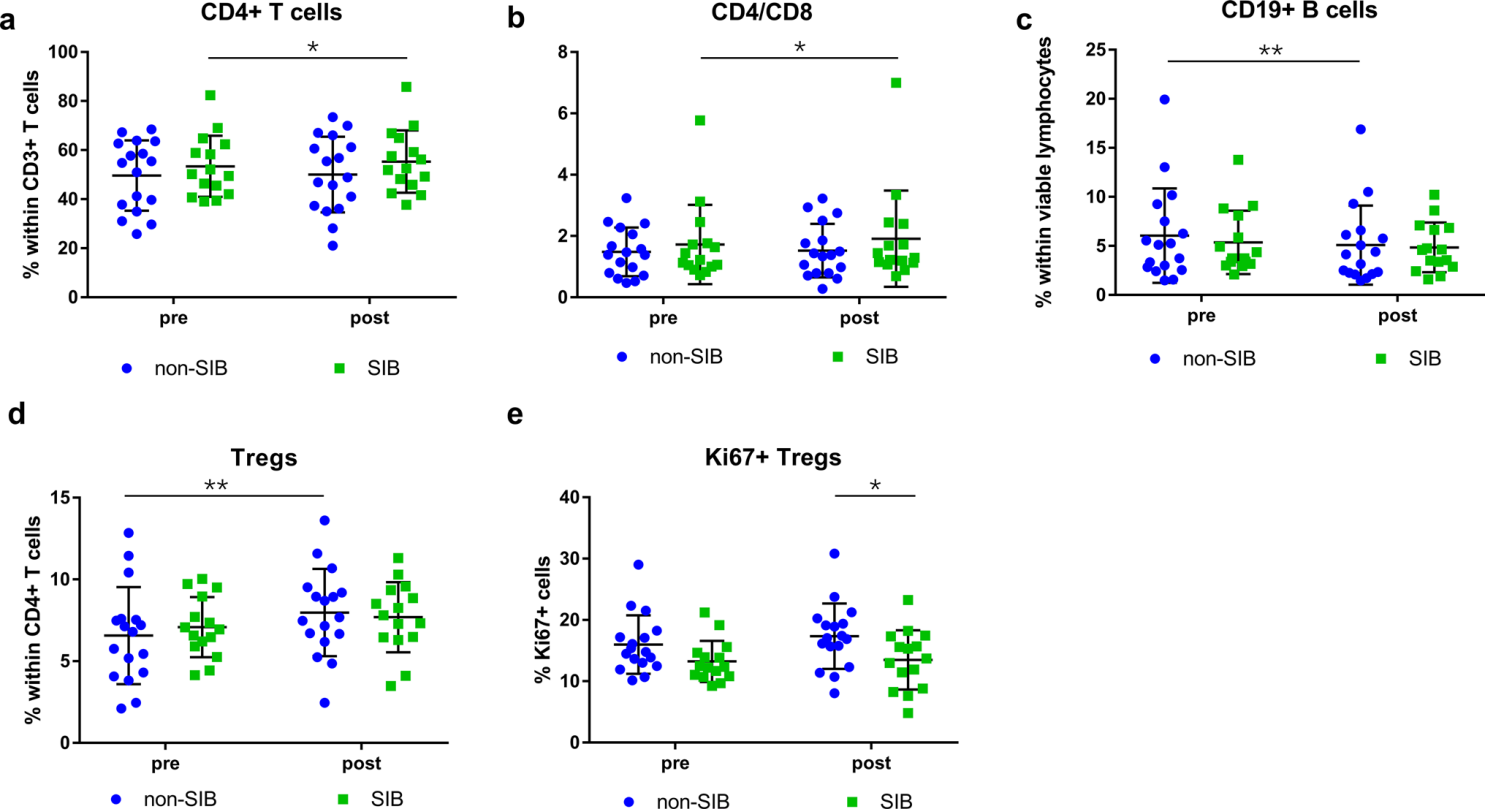
Immune changes after CIRT with or without SIB. Comparison of patients treated with CIRT with or without SIB (*N* = 15 or 17, respectively) for (**a**) percentage of CD4 + T cells within CD3 + T cells, (**b**) CD4/CD8 ratio, (**c**) CD19 + B cells within viable lymphocytes, (**d**) Tregs within CD4 + T cells, and (**e**) Ki67 + cells within Tregs. Significant differences are depicted as *p < 0.05 and **p < 0.01

To explore whether changes in immune cells are dependent on irradiation dose, we evaluated two patient groups separately, i.e. patients treated with CIRT of 65.6 GyE only (non-SIB group) and patients receiving additional SIB up to 72 GyE (SIB group). We observed an increase of the CD4 + T-cell percentage (pre: 53.4 ± 12.5%, post: 55.3 ± 12.7%; *P* = 0.0349) and CD4/CD8 ratio (pre: 1.72 ± 1.29, post: 1.91 ± 1.57; *P* = 0.0199) in the SIB group (Fig. 7a, b), while decreased CD19 + B cells (pre: 5.4 ± 3.2%, post: 4.8 ± 2.5%; *P* = 0.0021) and increased Tregs (pre: 7.1 ± 1.8%, post: 7.7 ± 2.1%; *P* = 0.0013) were detected in the non-SIB group (Fig. 7c, d). There was no statistically significant difference between the two groups for frequencies of all immune cell subsets (data not shown), however the proliferation of Tregs was significantly lower in the SIB versus non-SIB group after CIRT (17.4 ± 5.3% vs 13.5 ± 4.8%; *P* = 0.0411) (Fig. 7e).

## Discussion

Carbon ion radiotherapy is termed as an attractive radiotherapy approach based on improved therapeutic effect and decreased normal tissue toxicity. Radiotherapy-induced immunomodulation is closely related to the efficacy of radiotherapy and has gained extensive attention in multiple conventional photon radiotherapy studies. However, the impact of CIRT on the immune status of cancer patients is still not enough, especially prostate cancer. Therefore, the aim of this study was to investigate the immune response evoked by CIRT in localized prostate cancer patients, from the perspective of changes in the immune cell subsets and related cytokines with proinflammatory or immunosuppressive properties.

In this study, peripheral blood lymphocytes including CD3 + T, CD4 + T, CD8 + T and NK cells did not significantly decrease after CIRT, suggesting that CIRT caused negligible damage to peripheral lymphocytes. This lymphocyte protective effect may be attributed to the presence of Bragg peak and superior dose distribution of carbon ion beams. Spina et al. (Spina et al. 2021) also reported that low-dose CIRT may spare lymphocytes which were critical to the development of an adaptive anti-tumor immune response, while photon therapy was lymphotoxic even at low doses. However, CIRT indeed caused a drop in CD19 + B cells in our study, and this finding was in line with published data (Belka et al. 1999) describing that B lymphocytes were most vulnerable to local radiotherapy. Actually, CD4/CD8 ratio was a sensitive and stable marker of anti-tumor cellular immunity (Yang et al. 2016) and a higher CD4/CD8 ratio was related to better distant metastasis-free survival in patients with nasopharyngeal carcinoma treated by intensity modulated radiotherapy (Tao et al. 2016). We observed an increased CD4/CD8 ratio in our research, suggesting that CIRT was likely to improve anti-tumor immunity and be associated with better prognosis for localized prostate cancer patients. Of note, all the lymphocyte subsets mentioned above displayed a significantly increased proliferation rate after CIRT.

In addition to increased proliferation, peripheral CD4 + T cells and CD8 + T cells exhibited increased functionality after CIRT, characterized by modestly increased cytokine secretion of TNF. Increased inflammatory cytokine level of TNF-α can promote the killing of cancer cells and amplify the proliferation of anti-tumor immune cells like CD8 + T cells and NK cells (Mortezaee and Najafi 2021). However, Eckert F et al. (Eckert et al. 2018) did not observe enhanced T-cell function featured with increased effector cytokines after curative radiotherapy in prostate cancer patients, despite the small sample size and the use of superantigen Staphylococcus Enterotoxin B as a stimulant. In an orthotopic mammary tumor model (Spina et al. 2021), CD8 + T cells displayed increased production of granzyme B, IL-2, and TNF-a at higher doses of CIRT while high-dose photon therapy did not induce secretion of these cytokines from CD8 + tumor-infiltrating lymphocytes. These data and our results indicated that CIRT activated immune responses by inducing CD8 + T cells and CD4 + T cells to be reprogrammed into more functional cells, which might be expected to translate into better treatment response for prostate cancer patients treated by CIRT.

MDSCs are a heterogeneous population of immature cells of myeloid origin (Lin et al. 2021) and have been recognized to play a major role in suppression of both the innate and adaptive immunity. Increased frequencies of circulating M-MDSC in prostate cancer patients were associated with known negative prognostic markers including elevated level of prostate-specific antigen and shorter median overall survival (Idorn et al. 2014). Preclinical study (Xu et al. 2013) demonstrated that radiotherapy of prostate cancer induced a systemic increase of MDSCs in spleen, lung, lymph nodes and peripheral blood. Besides, clinical data (Nickols et al. 2021) observed a shift towards myeloid cell infiltration drived by stereotactic body radiotherapy in localized high-risk prostate cancer. We did not note a significant increase in M-MDSCs and E-MDSCs after CIRT, indicating of limited induction of immunosuppressive MDSCs, which might preserve the desired anti-tumor immunity, and favorable tendency to overcome MDSC-related radioresistance post-CIRT.

Tregs are a subset of T cells known for their immunosuppressive properties, which are important for maintaining immune homeostasis and preventing autoimmune diseases (Sakaguchi 2005). Cancer patients with aggregation of circulating or tumor- infiltrating Tregs tended to have a poor prognosis (Kotsakis et al. 2016). We observed an increase in the percentage of Tregs after CIRT, accompanied by unaffected proliferation of Tregs. Besides, Spina CS et al. found (Spina et al. 2021) that high-dose CIRT significantly increased the abundance but no proliferation of Tregs in the tumor microenvironment (TME). Targeting the immunosuppressive Tregs might provide a promising strategy to enhance the immunostimulatory effects of CIRT in patients with prostate cancer. However, a few studies have found that high Treg infiltration was related to a favorable prognosis in several cancer types, such as colorectal cancer (Correale et al. 2010) and non-small cell lung cancer (Koh et al. 2020). One explanation for this unusual finding was that Treg expansion relied on IL-2 levels produced by specific antigen-activated effector lymphocytes either CD4 + or CD8 + T cells (Sojka et al. 2008; Romano et al. 2019) to weaken a potentially harmful overreactive immune response, although no significant increase in the level of IL-2 expression was observed in prostate cancer patients receiving CIRT in our study, indicating that other mechanisms like the binding of TNF to TNF receptor type 2 might contribute to expansion of Tregs (Salomon et al. 2018). Based on this, high levels of Tregs in circulation as well as TME were inferred to be indirect indicators of anti-tumor immune response. Therefore, larger patient cohorts and long-term follow-up outcomes are urgently needed to validate the role of Treg as a predictive marker for therapy response and prognosis in prostate cancer patients treated with CIRT.

According to our results, TGF-β1 gene expression decreased significantly while IL-6 expression showed a downward trend after CIRT. Bouquet et al. (Bouquet et al. 2011) suggested that TGF-β inhibition was associated with increased sensitivity to radiotherapy and might be an effective adjunct in cancer radiotherapy. In addition, IL-6 inhibition was a potential therapeutic strategy for increasing radiosensitivity of prostate cancer (Wu et al. 2013). We then proposed that prostate cancer patients tended to benefit from CIRT, as evidenced by decreased TGF-β and IL-6. In surprise, we did not find any significant correlation between TGF-β1 gene expression from PBMCs and protein levels from plasma, probably due to that plasma contained TGF-β1 secreted by various cell subtypes such as stromal cells other than Tregs and MDSCs (Dahmani and Delisle 2018).

Considering a quarter of prostate cancer patients who underwent definitive radiotherapy may experience recurrence after treatment, increasing irradiation dose is of great significance for improving the efficacy of prostate cancer. A meta-analysis (Viani et al. 2008) suggested that every 1-Gy increase in irradiation dose may reduce the risk of biochemical relapse by approximately 1.8%. Referring to the fact that the most common local recurrence site of prostate cancer was the primary macroscopic tumor (Pucar et al. 2007), simultaneous integrated boost to the intraprostatic lesion has been explored and proven to be a practical technique to increase efficacy without increasing the toxicities (Kerkmeijer et al. 2021). Actually, the immunomodulatory effect of radiotherapy is related to the radiation dose. There was evidence that both photon and carbon ion irradiation could increase the surface expression of immunomodulatory molecules PD-L1, CD73, H2-Db and H2-Kb and the susceptibility of tumor cells to cytotoxic T cell-mediated cytolysis in a dose-dependent manner (Hartmann et al. 2020). In this study, we observed an increase of the CD4 + T cells and CD4/CD8 ratio in the SIB group but not in the non-SIB group after CIRT, suggesting that higher doses had the potential to induce more powerful tumor cytotoxicity and tumor antigen release, which were beneficial to CD4 + T-cell activation and expansion. There was no significant reduction in lymphocyte subsets in the SIB group compared with the non-SIB group, indicating that simultaneous integrated boost to macroscopic visible tumor was safe as it did not cause additional damage to lymphocyte subpopulations. From an immunologic perspective, these results supported the efficacy and safety of CIRT with SIB in the treatment of prostate cancer.

It is noteworthy that the majority (30/32 patients) of prostate cancer patients treated with CIRT in this study received concurrent hormonal therapy, in which case the effect of hormonal therapy on the immune system should not be ignored. Whereas androgen has been implicated as a negative regulator of host immune function, androgen deprivation therapy (ADT) was proven to increase levels of peripheral T cells and cause more vigorous antigen-specific T-cell proliferation in normal tumor-free mice (Roden et al. 2004). Indeed, hormone therapy could trigger apoptosis of hormone-dependent tumor cells, recruitment of antigen-presenting cells and infiltration of T cells in the prostate (Mercader et al. 2001). Additionally, Wu CT et al. (Wu et al. 2018) observed a longer radiotherapy-induced tumor growth delay associated with increased tumor-infiltrating T cells and attenuated MDSC recruitment in ADT-treated mice compared to those without ADT, and suggested that ADT enhanced radiotherapy sensitivity through immune-mediated mechanism. Based on these data, we suggest that the immune response evoked by CIRT could be modulated by hormonal therapy in our study.

It is reasonable to speculate that partial immunomodulatory effects after treatment might be the underlying mechanism by which CIRT overcomes radioresistance and exerts a positive effect on tumor control and clinical prognosis for patients with localized prostate cancer. Nevertheless, key limitations of this study that need to be taken seriously include lack of long-term clinical outcome, which is quite important to confirm the role of peripheral immunological parameters as biomarkers to predict outcome and therapy response. Another drawback includes a relatively small sample size and we are going to expand our study to more patients. It is worth mentioning that peripheral immune changes might not fully reflect intratumoral situation, so that immunologic effects in the TME of prostate cancer after CIRT and in-depth mechanism remain to be elucidated.

In conclusion, our study provides evidence of the immune response evoked by CIRT in localized prostate cancer patients. We found that CIRT may induce immune activation based on sparing lymphocytes, increased lymphocyte proliferation, enhanced T-cell functionality, together with limited induction of immunosuppressive cells and reduced expression of immunosuppressive cytokines. We expect that our study will provide a preliminary basis for attempts to combine CIRT with immunotherapy to increase tumor control and improve prognosis in prostate cancer. Further longitudinal study with a larger sample size is clearly warranted.

## Supplementary Information

Below is the link to the electronic supplementary material.

Supplementary file1 (DOCX 411 KB)

## Data Availability

The datasets generated during and/or analyzed during the current study are available from the corresponding author on reasonable request.

## References

[CR1] Belka C, Ottinger H, Kreuzfelder E, Weinmann M, Lindemann M, Lepple-Wienhues A (1999). Impact of localized radiotherapy on blood immune cells counts and function in humans. Radiother Oncol.

[CR2] Bouquet F, Pal A, Pilones KA, Demaria S, Hann B, Akhurst RJ (2011). TGFβ1 inhibition increases the radiosensitivity of breast cancer cells in vitro and promotes tumor control by radiation in vivo. Clin Cancer Res.

[CR3] Bronte V, Zanovello P (2005). Regulation of immune responses by L-arginine metabolism. Nat Rev Immunol.

[CR4] Correale P, Rotundo MS, Del Vecchio MT, Remondo C, Migali C, Ginanneschi C (2010). Regulatory (FoxP3+) T-cell tumor infiltration is a favorable prognostic factor in advanced colon cancer patients undergoing chemo or chemoimmunotherapy. J Immunother.

[CR5] Dahmani A, Delisle JS (2018). TGF-β in T cell biology: implications for cancer immunotherapy. Cancers.

[CR6] Demaria S, Golden EB, Formenti SC (2015). Role of local radiation therapy in cancer immunotherapy. JAMA Oncol.

[CR7] Deng L, Liang H, Xu M, Yang X, Burnette B, Arina A (2014). STING-dependent cytosolic DNA sensing promotes radiation-induced type I interferon-dependent antitumor immunity in immunogenic tumors. Immunity.

[CR8] Durante M, Formenti SC (2018). Radiation-induced chromosomal aberrations and immunotherapy: micronuclei, cytosolic DNA, and interferon-production pathway. Front Oncol.

[CR9] Eckert F, Schaedle P, Zips D, Schmid-Horch B, Rammensee HG, Gani C (2018). Impact of curative radiotherapy on the immune status of patients with localized prostate cancer. Oncoimmunology.

[CR10] Gabrilovich DI, Nagaraj S (2009). Myeloid-derived suppressor cells as regulators of the immune system. Nat Rev Immunol.

[CR11] Hartmann L, Schröter P, Osen W, Baumann D, Offringa R, Moustafa M (2020). Photon versus carbon ion irradiation: immunomodulatory effects exerted on murine tumor cell lines. Sci Rep.

[CR12] Idorn M, Køllgaard T, Kongsted P, Sengeløv L, Thor Straten P (2014). Correlation between frequencies of blood monocytic myeloid-derived suppressor cells, regulatory T cells and negative prognostic markers in patients with castration-resistant metastatic prostate cancer. Cancer Immunol Immunother.

[CR13] Kalbasi A, June CH, Haas N, Vapiwala N (2013). Radiation and immunotherapy: a synergistic combination. J Clin Invest.

[CR14] Kerkmeijer LGW, Groen VH, Pos FJ, Haustermans K, Monninkhof EM, Smeenk RJ (2021). Focal boost to the intraprostatic tumor in external beam radiotherapy for patients with localized prostate cancer: results from the FLAME randomized phase III trial. J Clin Oncol.

[CR15] Koh J, Hur JY, Lee KY, Kim MS, Heo JY, Ku BM (2020). Regulatory (FoxP3(+)) T cells and TGF-β predict the response to anti-PD-1 immunotherapy in patients with non-small cell lung cancer. Sci Rep.

[CR16] Kotsakis A, Koinis F, Katsarou A, Gioulbasani M, Aggouraki D, Kentepozidis N (2016). Prognostic value of circulating regulatory T cell subsets in untreated non-small cell lung cancer patients. Sci Rep.

[CR17] Liang H, Deng L, Hou Y, Meng X, Huang X, Rao E (2017). Host STING-dependent MDSC mobilization drives extrinsic radiation resistance. Nat Commun.

[CR18] Lin L, Kane N, Kobayashi N, Kono EA, Yamashiro JM, Nickols NG (2021). High-dose per fraction radiotherapy induces both antitumor immunity and immunosuppressive responses in prostate tumors. Clin Cancer Res.

[CR19] Liu W, Putnam AL, Xu-Yu Z, Szot GL, Lee MR, Zhu S (2006). CD127 expression inversely correlates with FoxP3 and suppressive function of human CD4+ T reg cells. J Exp Med.

[CR20] Martin NE, D'Amico AV (2014). Progress and controversies: radiation therapy for prostate cancer. CA Cancer J Clin.

[CR21] Mercader M, Bodner BK, Moser MT, Kwon PS, Park ES, Manecke RG (2001). T cell infiltration of the prostate induced by androgen withdrawal in patients with prostate cancer. Proc Natl Acad Sci USA.

[CR22] Mohamad O, Tabuchi T, Nitta Y, Nomoto A, Sato A, Kasuya G (2019). Risk of subsequent primary cancers after carbon ion radiotherapy, photon radiotherapy, or surgery for localised prostate cancer: a propensity score-weighted, retrospective, cohort study. Lancet Oncol.

[CR23] Mortezaee K, Najafi M (2021). Immune system in cancer radiotherapy: resistance mechanisms and therapy perspectives. Crit Rev Oncol Hematol.

[CR24] Nickols NG, Ganapathy E, Nguyen C, Kane N, Lin L, Diaz-Perez S (2021). The intraprostatic immune environment after stereotactic body radiotherapy is dominated by myeloid cells. Prostate Cancer Prostatic Dis.

[CR25] Pucar D, Hricak H, Shukla-Dave A, Kuroiwa K, Drobnjak M, Eastham J (2007). Clinically significant prostate cancer local recurrence after radiation therapy occurs at the site of primary tumor: magnetic resonance imaging and step-section pathology evidence. Int J Radiat Oncol Biol Phys.

[CR26] Roden AC, Moser MT, Tri SD, Mercader M, Kuntz SM, Dong H (2004). Augmentation of T cell levels and responses induced by androgen deprivation. J Immunol.

[CR27] Romano M, Fanelli G, Albany CJ, Giganti G, Lombardi G (2019). Past, present, and future of regulatory T cell therapy in transplantation and autoimmunity. Front Immunol.

[CR28] Sakaguchi S (2005). Naturally arising Foxp3-expressing CD25+CD4+ regulatory T cells in immunological tolerance to self and non-self. Nat Immunol.

[CR29] Salomon BL, Leclerc M, Tosello J, Ronin E, Piaggio E, Cohen JL (2018). Tumor necrosis factor α and regulatory T cells in oncoimmunology. Front Immunol.

[CR30] Sato H, Kasuya G, Ishikawa H, Nomoto A, Ono T, Nakajima M (2021). Long-term clinical outcomes after 12-fractionated carbon-ion radiotherapy for localized prostate cancer. Cancer Sci.

[CR31] Schaue D, Xie MW, Ratikan JA, McBride WH (2012). Regulatory T cells in radiotherapeutic responses. Front Oncol.

[CR32] Siegel RL, Miller KD, Fuchs HE, Jemal A (2021). Cancer statistics, 2021. CA Cancer J Clin.

[CR33] Sojka DK, Huang YH, Fowell DJ (2008). Mechanisms of regulatory T-cell suppression - a diverse arsenal for a moving target. Immunology.

[CR34] Spina CS, Tsuruoka C, Mao W, Sunaoshi MM, Chaimowitz M, Shang Y (2021). Differential immune modulation with carbon-ion versus photon therapy. Int J Radiat Oncol Biol Phys.

[CR35] Takakusagi Y, Katoh H, Kano K, Anno W, Tsuchida K, Mizoguchi N (2020). Preliminary result of carbon-ion radiotherapy using the spot scanning method for prostate cancer. Radiat Oncol.

[CR36] Tao CJ, Chen YY, Jiang F, Feng XL, Jin QF, Jin T (2016). A prognostic model combining CD4/CD8 ratio and N stage predicts the risk of distant metastasis for patients with nasopharyngeal carcinoma treated by intensity modulated radiotherapy. Oncotarget.

[CR37] Viani GA, Stefano EJ, Afonso SL (2008). Higher-than-conventional radiation doses in localized prostate cancer treatment: a meta-analysis of randomized, controlled trials. Int J Radiat Oncol Biol Phys.

[CR38] Wu CT, Chen MF, Chen WC, Hsieh CC (2013). The role of IL-6 in the radiation response of prostate cancer. Radiat Oncol.

[CR39] Wu CT, Hsieh CC, Yen TC, Chen WC, Chen MF (2015). TGF-β1 mediates the radiation response of prostate cancer. J Mol Med.

[CR40] Wu CT, Chen WC, Chen MF (2018). The response of prostate cancer to androgen deprivation and irradiation due to immune modulation. Cancers.

[CR41] Xia C, Dong X, Li H, Cao M, Sun D, He S (2022). Cancer statistics in China and United States, 2022: profiles, trends, and determinants. Chin Med J.

[CR42] Xu J, Escamilla J, Mok S, David J, Priceman S, West B (2013). CSF1R signaling blockade stanches tumor-infiltrating myeloid cells and improves the efficacy of radiotherapy in prostate cancer. Cancer Res.

[CR43] Yang ZR, Zhao N, Meng J, Shi ZL, Li BX, Wu XW (2016). Peripheral lymphocyte subset variation predicts prostate cancer carbon ion radiotherapy outcomes. Oncotarget.

