# Supplementary material for: Immunomodulatory effects of carbon ion radiotherapy in patients with localized prostate cancer
Source: J Cancer Res Clin Oncol. 2022 Sep 23;149(8):4533–45. doi: 10.1007/s00432-022-04194-9 (PMC10349746; doi:10.1007/s00432-022-04194-9)
Supplement: Supplementary file 1 — Supplementary file1 (DOCX 411 KB) [file 432_2022_4194_MOESM1_ESM.docx]

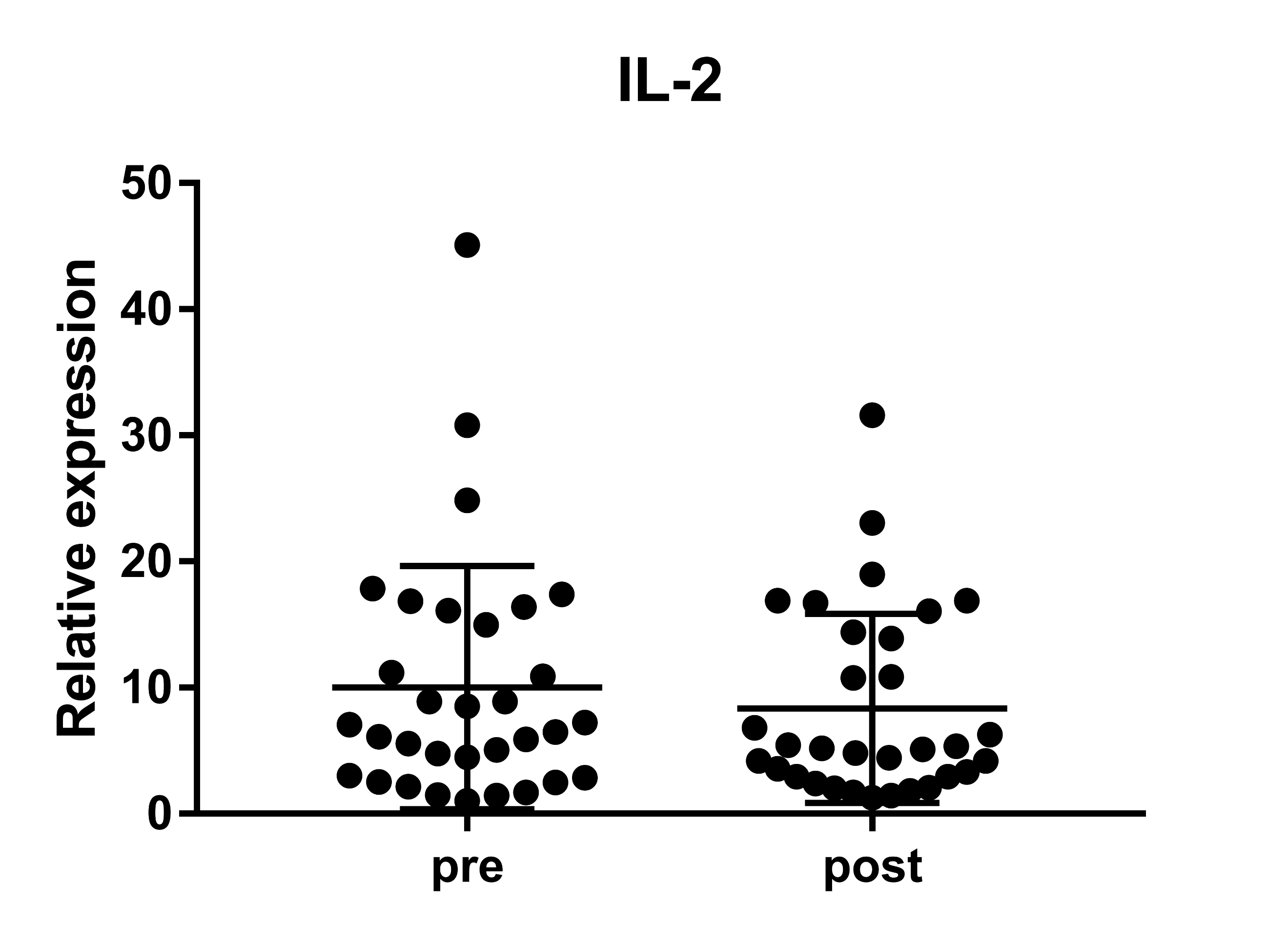


**Supplementary Fig. 1** Cytokine gene expression level of IL-2 is shown before and after CIRT. N=32 CIRT patients are included
